# Cushing's syndrome due to ectopic ACTH production by a nasal paraganglioma

**DOI:** 10.1530/EDM-13-0038

**Published:** 2013-09-16

**Authors:** F Serra, S Duarte, S Abreu, C Marques, J Cassis, M Saraiva

**Affiliations:** Department of EndocrinologyHospital Egas Moniz, Centro Hospitalar Lisboa OcidentalLisbonPortugal; 1 Department of EndocrinologyHospital Central do FunchalFunchalPortugal; 2 Department of NeurosurgeryHospital Egas Moniz, Centro Hospitalar Lisboa OcidentalLisbonPortugal; 3 Department of PathologyHospital Egas Moniz, Centro Hospitalar Lisboa OcidentalLisbonPortugal

## Abstract

Ectopic secretion of ACTH is an infrequent cause of Cushing's syndrome. We report a case of ectopic ACTH syndrome caused by a nasal paraganglioma, a 68-year-old female with clinical features of Cushing's syndrome, serious hypokalaemia and a right paranasal sinus' lesion. Cranial magnetic resonance image showed a 46-mm mass on the right paranasal sinuses. Endocrinological investigation confirmed the diagnosis of ectopic ACTH production. Resection of the tumour normalised ACTH and cortisol secretion. The tumour was found to be a paraganglioma through microscopic analysis. On follow-up 3 months later, the patient showed nearly complete clinical recovery. Ectopic ACTH syndrome due to nasal paraganglioma is extremely uncommon, as only two other cases have been discussed in the literature.

**Learning points:**

- Ectopic Cushing's syndrome accounts for 10% of Cushing's syndrome etiologies.
- Most paraganglioma of the head and neck are not hormonally active.
- Nasal paraganglioma, especially ACTH producing, is a very rare tumour.

## Background

Paragangliomas are highly vascular tumours derived from the embryonic neural crest. Although they are benign in the majority of cases, they can lead to a clinical presentation when they are hormonally active. The clinical manifestation depends on the hormone produced by the paraganglioma and includes Cushing's syndrome (adrenocorticotropic hormone (ACTH)), hypercalcaemia (parathyroid-related peptide), inappropriate secretion of antidiuretic hormone syndrome (antidiuretic hormone), diarrhoea (intestinal vasoactive peptide) and acromegaly (growth hormone) (1).

Paragangliomas occur most commonly at the carotid bifurcation in the head and neck where they are referred to as carotid body tumours. Other common sites of origin include the jugular bulb (jugular paraganglioma (JP)), the tympanic plexus on the promontory (tympanic paraganglioma (TP)) and the vagal nerve (vagal paraganglioma). They are very unusual in the paranasal sinuses. Patients with cervical paragangliomas frequently present with a painless, slow-growing mass in the lateral neck. In many patients with TP and JP, tinnitus and hearing loss are early symptoms. The paragangliomas located on the head and neck mostly do not secrete catecholamines or metanephrines (2).

Cushing's syndrome is a disorder of hypercortisolism which is characterised by moon facies, buffalo hump, truncal obesity and purple striae. It is associated with cardiovascular, endocrine, neural, gastrointestinal, skin and musculoskeletal dysfunction. Ectopic secretion of ACTH accounts for 10% of Cushing's syndrome etiologies (3). Ectopic ACTH syndrome due to nasal paraganglioma is extremely rare; there have been report of fewer than three cases in the literature (4) (5).

## Case presentation

A case of a 68-year-old woman with a previous history of controlled hypertension and sinusitis developed symptoms of progressive hearing loss over the past year. She also complained of leg oedema and started taking furosemide. At that time, the laboratory results showed hyperglycaemia. One year later, she presented to the emergency department with dizziness, tiredness, polyuria and polydipsia. On physical examination, the patient had mildly elevated blood pressure (150/79 mmHg) and appeared severely cushingoid, with hyperpigmentation and facial rounding, purple striae on the abdomen, scattered bruises and central obesity. Laboratory results revealed glycaemia >200 mg/dl, high white blood cell count with high neutrophils and severe hypokalaemia (1.9 mmol/dl). Cushing's syndrome was suspected and endocrine evaluation was done.

## Investigation

The blood results revealed highly elevated ACTH and cortisol levels (ACTH, 317.0 pg/ml (<46); AM cortisol, 98.7 μg/dl (5–25.0) and 24-h urine cortisol, 1770.5 μg/24 h total volume (20–90)). Dexamethasone suppression test was considered: however, in the presence of very high ACTH and cortisol levels, hypokalaemia, as well as clinical findings, a primary pituitary tumour or an ectopic ACTH syndrome was suspected, so the test was not performed.

**Figure 1:**
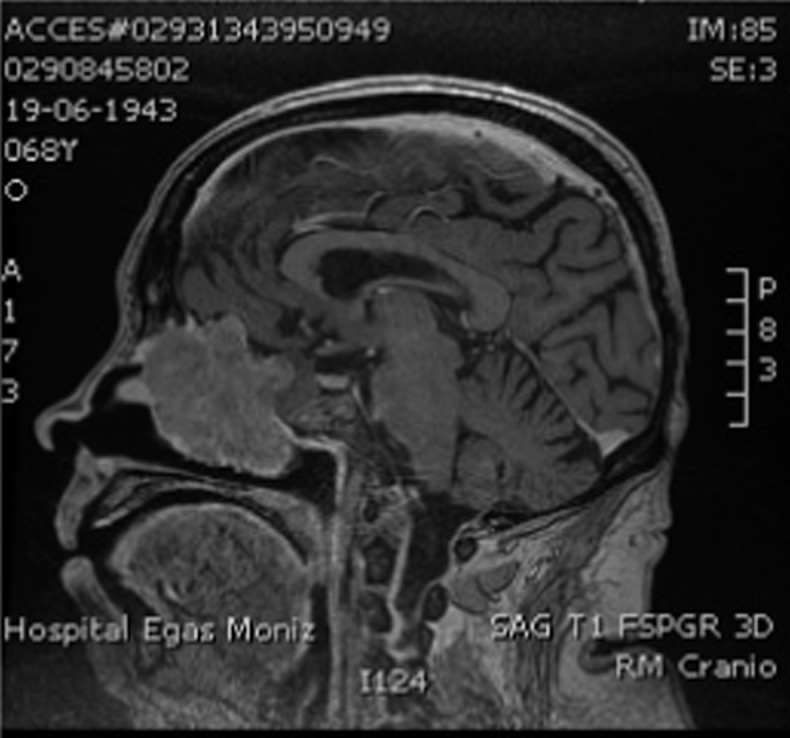
Cranial MRI showing a large mass on the right nasal sinuses.

A pituitary magnetic resonance image (MRI) showed a normal pituitary; however, there was a large, aggressive mass with probable origin on the right nasal sinuses occupying globally the ethmoid as well (Fig. 1). The cribiform plate was eroded focally, with nearly 15 mm extended into the frontal sinus. However, the mass did not exceed 6 mm into the frontal lobe and had no repercussion there. The mass also extended to the sphenoid sinus but spared the maxillary sinuses. The lesion's size was 44 mm coronal, 46 mm sagital and 15 mm axial.

She was then sent to our centre for evaluation. After the diagnosis of ectopic ACTH syndrome, she started taking ketoconazle 200 mg, four times daily (total dose of 800 mg/day).

Computed tomography scan of the chest and abdomen were normal except for hyperplastic adrenal glands. Bone density scan (dual energy X-ray absorptiometry (DEXA)) showed osteopaenia of the spine and osteoporosis of the hip with a *T*-score of −3.

The MR appearance of the mass, along with the clinical and laboratory findings, was suspicious for esthesioneuroblastoma. Inferior petrosal deep venous catheterisation was not performed due to highly suspected ectopic Cushing's syndrome in relation to this mass. The patient consulted with an otolaryngologist and a biopsy was performed. Despite the small amount obtained the result showed focal expression of ACTH and chromogranin A and the diagnosis of ACTH-secreting esthesioneuroblastoma was assumed. She was referred to surgery.

## Treatment

To control her metabolic abnormalities, the patient was treated with ketoconazole 200 mg, four times daily (total dose of 800 mg/day). Two weeks later octreotide (0.1 mg) three times a day was added. The patient's cortisol concentration dropped with this treatment, but she had severe hypokalaemia that was difficult to control and was under high doses of potassium (360 mEq/day) and spironolactone (200 mg/day). Ten days after the start of ketoconazole, her cortisol level was 51.3 μg/dl (5–25.0); 24-h urine cortisol, 1202 μg/24-h total volume (20–90) and ACTH, 235 pg/ml (<46) and 3 days after the initiation of octreotide, cortisol levels were 48 μg/dl. Owing to hypothyroidism (THS, 0.02 μU/ml (0.46–4.68) and free thyroxine, 8.27 pmol/l (10–28.2)), in relation to high ACTH, she started levothyroxine 0.05 mg. She also required diuretics, calcium, insulin, magnesium and albumin.

Bilateral pneumonia due to MRSA and respiratory distress occurred due to her immunosuppressive condition. She was transferred to the Intensive Unity Care and the surgery to remove the tumour was delayed. Twenty-five days after starting the treatment with ketoconazole, she developed toxic hepatitis. As metyrapone was not available and the surgery was planned, ketoconazole was stopped. Progressively liver function returned to normal. After recovery of the pulmonary symptoms, 1 month after her initial presentation in our centre, the patient underwent an approach to the anterior cranial fossa. Gross total resection was performed in a combined effort by the neurosurgery and otolaryngology services. The patient was treated with a stress dose of steroids postoperatively. Grossly the tumour was soft with fragments between 3 and 12 mm.

**Figure 2:**
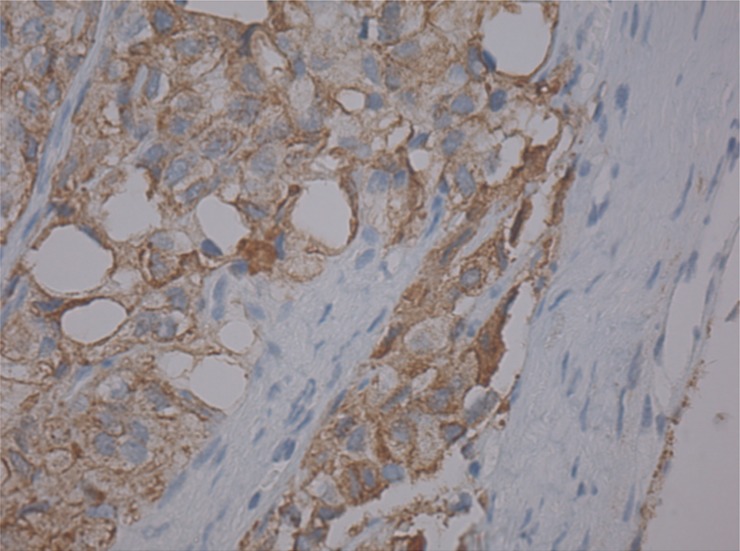
ACTH+ staining.

**Figure 3:**
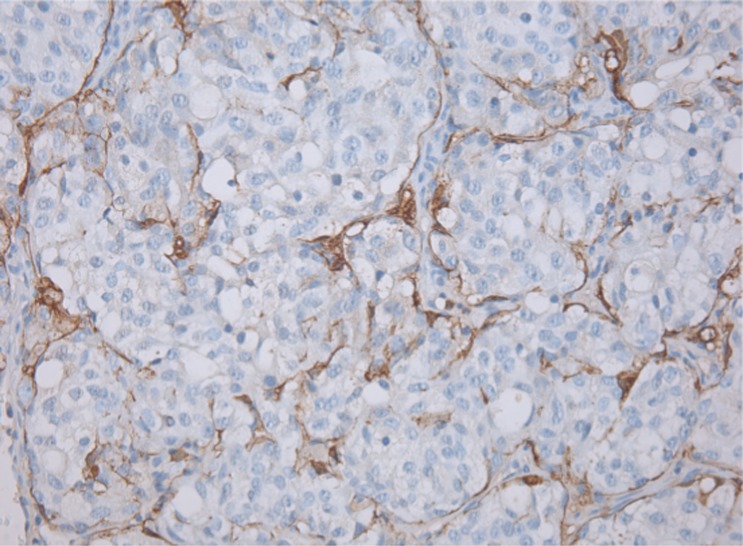
S-100+ staining.

**Figure 4:**
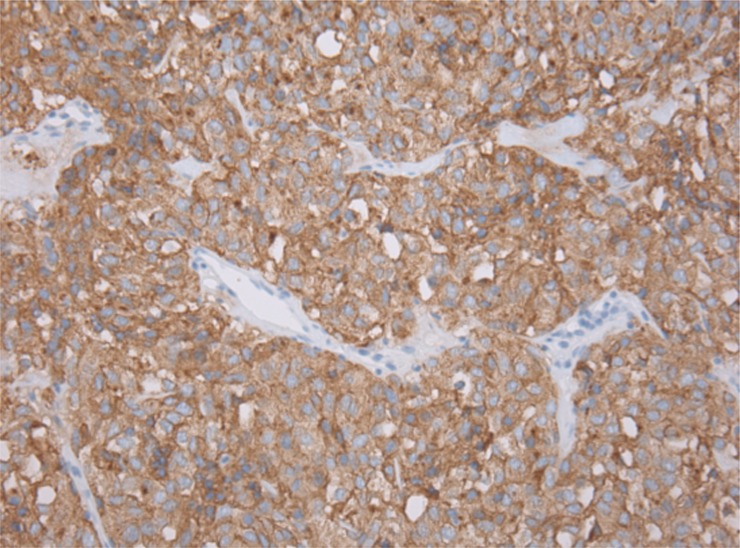
Synaptophysin+ staining.

Microscopically the tumour had lobular architecture with big, round cells and with eosinophilic granular cytoplasm. These cells were NSE+, synaptophysin+, CD56+, CD57+ and ACTH+. Surrounding the tumour lobules were several spindle cells S-100+ (sustentacular cells). These tumour cells were negative for FSH, TSH, GH, LH and prolactin (Figs 2, 3 and 4).

## Outcome and follow-up

**Figure 5:**
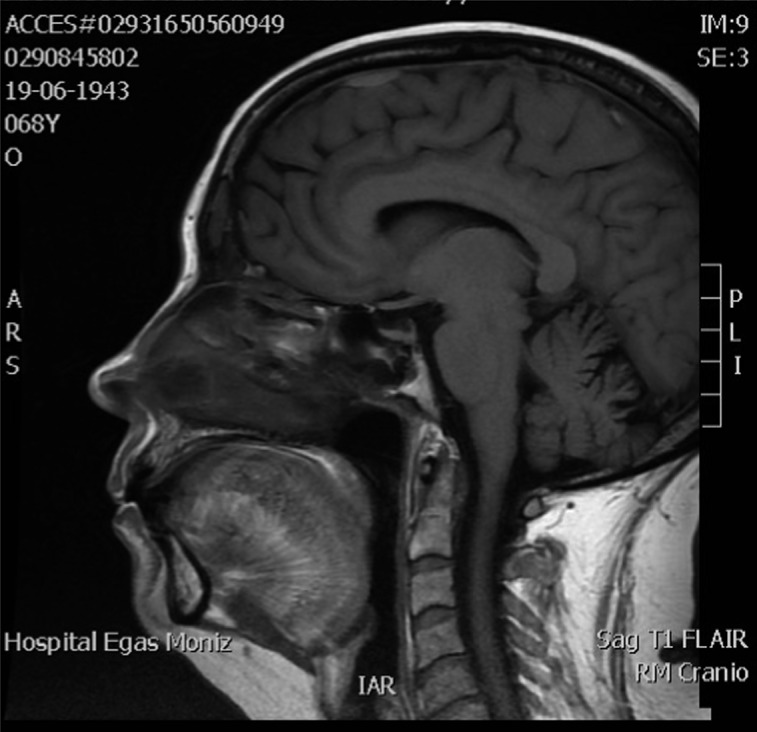
Post-operative cranial MRI.

Furthermore, the woman's Cushing's syndrome symptoms completely resolved. Two weeks after surgery, urinary free cortisol level was 57 μg/24 h total volume (20–90) and ACTH was 12.9 pg/ml (<46). Progressively clinical features of moon facies, plethora and purple striae disappeared. She lost weight and reported life quality improvement. An MRI 6 months postoperatively demonstrated no evidence of recurrence (Fig. 5).

## Discussion

The initial diagnosis of esthesioneuroblastoma was not confirmed after surgery. The immunohistochemical staining was important in establishing the diagnosis of nasal paraganglioma. The lobular growth pattern, the presence of sustentacular cells (S-100 positive) and the ACTH-producing granules favour an ACTH-producing nasal paraganglioma diagnosis.

Ectopic Cushing's syndrome is usually caused by small cell carcinomas of the lung, carcinoid tumours, pancreatic islet cell tumours, medullary thyroid carcinomas and rarely paragangliomas (3). Ectopic ACTH production is an uncommon manifestation of this uncommon disease. The first published case (4) in 1982 was in a patient with similar clinical features as our patient. He was treated with methyrapone, tumour resection and irradiation of the tumour which resulted in a lasting remission. In the second published case (5) in 2003, the patient was cured with tumour resection. We report the third case of ectopic ACTH production with nasal paraganglioma. In all cases, the characteristic alveolar pattern of a paraganglioma, the ACTH granules and the presence of sustentacular cells made the diagnosis clear.

The diagnosis of ectopic ACTH syndrome secondary to nasal paraganglioma was established based on the presence of ACTH seen on immunohistochemical staining of the tumour, the disappearance of symptoms, as well as decrease and normalisation of plasma ACTH and cortisol levels after resection of the tumour. Surgical total resection is the treatment of choice.

Around 5% of the paragangliomas of the head and neck are hormonally active and very rarely they secrete ACTH, which makes this case very unusual (1).

Owing to the high prevalence of mutations in the SDH gene in people suffering from paraganglioma, genetic testing is mandatory (6). When located on the head or neck the first genetic testing should be for SDHC (7). This patient is waiting for genetic testing which has been requested.

## Patient consent

Written informed consent was obtained from the patient for publication of this case report.

## Author contribution statement

S Duarte is an Endocrinologist Physician who followed the patient in the Endocrinology Department of Hospital Egas Moniz. S Abreu is also an Endocrinology Physician who followed the patient in Funchal, before the patient was sent to our department. C Marques is the Neurosurgeon who did the surgical procedure in Hospital Egas Moniz. J Cassis is the Pathologist who did the immunohistochemical staining of the tumour. M Saraiva is the director of the Endocrinology Department in Hospital Egas Moniz where the patient was treated.
